# Systemic inflammatory markers and volume of enhancing tissue on post-contrast T1w MRI images in differentiating true tumor progression from pseudoprogression in high-grade glioma

**DOI:** 10.1016/j.ctro.2024.100849

**Published:** 2024-08-30

**Authors:** Camilla Satragno, Irene Schiavetti, Eugenia Cella, Federica Picichè, Laura Falcitano, Martina Resaz, Monica Truffelli, Stefano Caneva, Pietro Mattioli, Daniela Esposito, Alessio Ginulla, Claudio Scaffidi, Pietro Fiaschi, Alessandro D’Andrea, Andrea Bianconi, Gianluigi Zona, Laura Barletta, Luca Roccatagliata, Lucio Castellan, Silvia Morbelli, Matteo Bauckneht, Isabella Donegani, Paolo Nozza, Dario Arnaldi, Giulia Vidano, Flavio Gianelli, Salvina Barra, Elisa Bennicelli, Liliana Belgioia, disease management team on Neuroncology of IRCCS Ospedale Policlinico San Martino

**Affiliations:** a Dept. of Experimental Medicine (DIMES), University of Genoa, Genoa, Italy; b Dept. of Health Science (DISSAL), University of Genoa, Genoa, Italy; c U.O. Oncologia Medica 2, IRCCS Ospedale Policlinico San Martino, Genoa, Italy; d Dept. of Internal Medicine and Medical Speciality (DIMI), University of Genoa, Genoa, Italy; f U.O. Neuroradiologia, IRCCS Ospedale Policlinico San Martino, Genoa, Italy; g U.O. Clinica Neurochirurgica e Neurotraumatologica, IRCCS Ospedale Policlinico San Martino, Genoa, Italy; h Department of Neuroscience Ophthalmological Rehabilitation Genetics and Mother and Child Health (DINOGMI), University of Genoa, Genoa, Italy; i U.O. Neurofisiopatologia, IRCCS Ospedale Policlinico San Martino, Genoa, Italy; j U.O. Medicina Nucleare, IRCCS Ospedale Policlinico San Martino, Genoa, Italy; k U.O. Anatomia Patologica Ospedaliera, IRCCS Ospedale Policlinico San Martino, Genoa, Italy; e U.O. Radioterapia Oncologica, IRCCS Ospedale Policlinico San Martino, Genoa, Italy

**Keywords:** High-grade glioma, Pseudoprogression, Systemic Immune-Inflammation Index, Neutrophil-Lymphocyte Ratio, Post-contrast T1-weighted volume, Volumetric analysis

## Abstract

**Background:**

High-grade glioma (HGG) patients post-radiotherapy often face challenges distinguishing true tumor progression (TTP) from pseudoprogression (PsP). This study evaluates the effectiveness of systemic inflammatory markers and volume of enhancing tissue on post-contrast T1 weighted (T1WCE) MRI images for this differentiation within the first six months after treatment.

**Material and Methods:**

We conducted a retrospective analysis on a cohort of HGG patients from 2015 to 2021, categorized per WHO 2016 and 2021 criteria. We analyzed treatment responses using modified RANO criteria and conducted volumetry on T1WCE and T2W/FLAIR images.

Blood parameters assessed included neutrophil/lymphocyte ratio (NLR), systemic immune-inflammation index (SII), and systemic inflammation response index (SIRI). We employed Chi-square, Fisher’s exact test, and Mann-Whitney *U* test for statistical analyses, using log-transformed predictors due to multicollinearity. A Cox regression analysis assessed the impact of PsP- and TTP-related factors on overall survival (OS).

**Results:**

The cohort consisted of 39 patients, where 16 exhibited PsP and 23 showed TTP. Univariate analysis revealed significantly higher NLR and SII in the TTP group [NLR: 4.1 vs 7.3, p = 0.002; SII 546.5 vs 890.5p = 0.009]. T1WCE volume distinctly differentiated PsP from TTP [2.2 vs 11.7, p < 0.001]. In multivariate regression, significant predictors included NLR and T1WCE volume in the “NLR Model,” and T1WCE volume and SII in the “SII Model.” The study also found a significantly lower OS rate in TTP patients compared to those with PsP [HR 3.97, CI 1.59 to 9.93, p = 0.003].

**Conclusion:**

Elevated both, SII and NLR, and increased T1WCE volume were effective in differentiating TTP from PsP in HGG patients post-radiotherapy. These results suggest the potential utility of incorporating these markers into clinical practice, though further research is necessary to confirm these findings in larger patient cohorts.

## Highlights

• Shows how increased tissue volume in post-contrast T1w MRI images predicts response to glioma radiotherapy.
• Links leukocyte count changes to glioma radiotherapy outcomes.
• Unveils reproducible, low-cost techniques for glioma detection via MRI and markers.

## 1 Introduction

High-grade gliomas (HGGs) are the most prevalent and aggressive primary malignant brain tumors in adults [1]. Despite advancements in molecular characterization, the prognosis, particularly for glioblastoma (GB) patients, remains bleak, with a low survival rate at one and five years post-treatments [2]. Since 2005, the standard of care has included maximal or supramaximal surgery, followed by radiotherapy (RT) and temozolomide (TMZ) chemotherapy [3], [4].

Accurately assessing treatment response and differentiating true tumor progression (TTP) from pseudoprogression (PsP) – a treatment-related transient image phenomenon – continues to pose significant challenge [5].

PsP, often resembling TTP on magnetic resonance imaging (MRI), results from treatment-related effects like radiation-induced inflammation and transient edema [6], [7].

Correctly distinguishing between PsP from TTP is crucial for informed treatment decisions and enhancing patient outcomes [8]. Misdiagnosing PsP as TTP can lead to unnecessary and potentially harmful interventions, while confusing TTP for PsP might delay crucial treatments [9]. Furthermore, accurate differentiation contributes to better patient management, improving quality of life by avoiding unnecessary procedures and the associated side effects of aggressive treatments [10].

Conventional MRI sequences have limited ability in accurately distinguishing between the two conditions [10]. Typically, PsP occurs within the first few months post-radiotherapy and chemotherapy, temporally overlapping with TTP imaging features [9].

To overcome this diagnostic challenge, several studies have explored advanced imaging modalities, such as positron emission tomography (PET) especially with amino acid tracers, magnetic resonance spectroscopy (MRS), perfusion-weighted imaging (PWI), diffusion-weighted imaging (DWI) and radiomic features, to provide additional differentiation insights [7], [8], [11], [12].

However, particularly within the first three months after treatment completion, these modalities have yet to reliably distinguish between PsP and TTP, often necessitating further examinations [12], [13].

Interestingly, increasing volume of enhancing tissue on post-contrast T1 weighted (T1WCE) has shown significant potential in predicting TTP [5]. Additionally, the analysis of genetic alterations and molecular markers, including O^6methylguanine-DNA methyltransferase (MGMT) promoter methylation status and isocitrate dehydrogenase (IDH) mutation presence, has demonstrated promise in enhancing diagnostic accuracy [8], [12], [14].

In recent years, identifying reliable, noninvasive blood biomarkers has gained attraction as potential markers for predicting treatment response and guiding therapeutic decisions in HGGs [15], [16], [17], [18], [19].

The blood cell-derived indices might complement tumor tissue-derived biomarkers, offering cost-effective and easily reproducible methods to improve prognostic stratification [20].

Inflammation, increasingly recognized as a pivotal factor in cancer progression, can be quantified using various indices [21] like the neutrophil–lymphocyte ratio (NLR), systemic immune-inflammation index (SII), and systemic inflammation response index (SIRI).

The NLR, a simple measure of systemic inflammation, has been extensively studied and is thought to reflect the balance between pro-tumor inflammation and anti-tumor immunity. Elevated NLR has been associated with poor prognosis in GB patients, correlating with shorter overall survival and progression-free survival [19], [22]. This suggests that patients with higher NLR may exhibit more aggressive tumor behavior and a diminished response to treatments.

Similarly, the SII, which incorporates platelet counts along with neutrophil and lymphocyte counts, has been demonstrated to have prognostic value in GB. Studies have indicated that patients with a higher SII tend to have a worse prognosis, potentially due to the role of platelets in promoting tumor growth and protecting circulating tumor cells [18], [21].

Lastly, the SIRI, which combines neutrophil, monocyte, and lymphocyte counts, is a newer index that has been shown to predict clinical outcomes in GB. A higher SIRI has been linked with a more immunosuppressive microenvironment, leading to poorer survival outcomes for patients [15], [20]. However, the utility of SIRI as a distinct prognostic tool compared to other indices is still under investigation.

While these indices show promise in enhancing our understanding of GB prognosis and treatment response, it’s important to note that their predictive power and clinical utility are not yet fully established, particularly in distinguishing between PsP and TTP, necessitating further research and validation in larger, prospective studies [16], [22], [23].

This study aims to integrate the systemic inflammatory indices and volume of enhancing tissue on post-contrast T1 weighted images to improve differentiation between PsP and TTP in HGG patients.

## 2 Materials and methods

This study was conducted following the approval from the ethical committee, under the protocol number 368/2021 – DB id 11595, ensuring adherence to ethical standards and guidelines for research.

### 2.1 Patient data

We conducted a retrospective cohort study from 2015 to 2021, enrolling patients with HGGs as classified by WHO 2016 criteria, adapted to the WHO 2021 standards, and by the WHO 2021 classification [24].

Clinical, MRI, and therapeutic data were collected at three distinct time points: at diagnosis, post-surgery or biopsy, and within six months post-RT (in most cases, PsP occurs within the first 3 months after completion of treatment but can occur up to 6 months after treatment [6]).

Inclusion criteria required:

• Integrated histopathological and molecular diagnosis of HGG,
• MRI follow-up suspected of PsP or TTP, within 6 months post-RT,
• Presence of a subsequent MRI to confirm PsP or TTP,
• A complete blood count at the same timing as MRI follow up. All parameters in the complete blood count, including Neutrophil, Lymphocyte, and Platelet counts, are measured in ×10^9 cells per liter (×10^9/L).

From the complete blood count, inflammation indices were analyzed: NLR (Neutrophils/Lymphocytes), SII (Platelet count x Neutrophil count/Lymphocyte count), and SIRI (Neutrophil count x Monocyte count)/Lymphocyte count).

Patient profiles were characterized by age and gender at the time of diagnosis.

The tumor sites were categorized based on diagnostic MRI, findings into two distinct categories, frontal lobe and other cerebral lobe, each bearing prognostic implications as referenced in studies [25], [26], [27], [28], [29], [30], [31]. Moreover, we accounted for the presence of multicentric disease at diagnosis.

Additionally, the administered corticosteroid dosages, standardized to dexamethasone equivalents exceeding 4 mg, were recorded during both the treatment phase and the subsequent follow-up period.

Surgical interventions for each patient were evaluated based on the residual disease post-operation: subtotal resection (STR) indicated the presence of residual disease, whereas gross total resection (GTR) denoted its absence. Cases where only a biopsy was performed were also noted.

We considered also the IDH mutation and the MGMT methylation status.

Furthermore, we took into account the presence of infections during and after radiotherapy.

Chemotherapy regimens were distinctly categorized based on their temporal relationship with radiotherapy, identifying whether they were administered concomitantly or sequentially.

### 2.2 Imaging data and response to treatment identification

Each MRI included standard sequences, (T1W, T1WCE, T2W and T2W/FLAIR).

The imaging was reviewed by a specialized neuroradiologists team.

MRIs were reviewed at diagnosis, within 72h post-surgery and during follow-ups.

Treatment response was evaluated using modified RANO criteria [32], then to define TTP and PsP we applied: mandatory confirmation of progression with a repeat MRI and measurement of the maximum tumour cross-sectional area with which we associated volumetric measurements.

Radiological patterns of relapse were defined as local or distal, considering growth of residual and/or new lesion.

### 2.3 Radiotherapy data

Patients were treated according to their performance status, age and extent of surgery, following protocols including the STUPP protocol [4] or alternatives for specific age and performance categories [33], [34].

Volumetry of suspected progression was performed on T1WCE and T2W/FLAIR sequences.

Three labels were identified, respectively, the suspected disease with contrast enhancement, the FLAIR hyperintensity indicative of perilesional oedema, and the whole tumour covering the whole FLAIR hyperintensity [35], [36], excluding the surgical cavity, where it was present.

Segmentation was conducted by our department's team of neuro-radiation oncologists using our Treatment Planning System (TPS).

### 2.4 Statistical analysis

Associations between clinicopathological factors and progression were assessed using Chi-square and Fisher's exact tests.

Continuous variables were analyzed using independent sample t-tests or Mann-Whitney U tests as appropriate.

Variables with a p-value ≤ 0.05 in univariate analysis were included in the multivariate logistic regression.

Logistic regression models were developed for prognostic factors of progression, with log-transformed predictors to address skewed distributions.

Two separate binary regression models addressed multicollinearity between the “NLR” and “SII index.”

In the “Multivariate Model NLR,” the SII index was not considered, while in the “Multivariate Model SII index,” the NLR was excluded.

Cox regression analysis evaluated the impact of PsP- and TTP-related factors on OS.

The discriminative capacity of significant variables was assessed using Receiver Operating Characteristic (ROC) curve analysis and Area Under the Curve (AUC) curve values. The curve comparison was carried out. The optimal threshold for maximizing sensitivity and specificity was identified, contributing to our diagnostic accuracy.

## 3 Results

The study included 39 patients out of initial cohort of 121, of 41.0% being females.

Among these, 16 patients exhibited PsP, while 23 showed TTP.

Of the 39 patients according to the WHO 2016 classification, 5 (12.8%) were grade 3 astrocytomas and 34 (87.2%) were glioblastomas. Based on molecular biology and thus transitioning to the 2021 classification, of the 5 considered grade 3, only 2 (40%) were confirmed as such, while the other 3 (60%) were reclassified as molecular glioblastomas due to the absence of the IDH gene mutation. Of the 34 considered glioblastomas, 1 (2.9%) presenting the IDH mutation was reclassified to IDH-mutant grade 4 astrocytoma [2].

The demographic and clinical characteristics, along with key imaging and blood test findings, are detailed in Table 1a, Table 1b, without showing notable differences between the two groups. In our cohort no patient was infected during and/or after the end of radiotherapy.

**Table 1a:** Part 1. Differences at baseline between TTP and PsP.

		TTP (N=23)	PsP (N=16)	*p*
sex	female	9 (39.1 %)	7 (43.8 %)	0.77
	male	14 (60.9 %)	9 (56.3 %)	
age at the time of diagnosis		61.1 ± 14.17	57.0 ± 14.62	0.39
cerebral lobe	other lobes	14 (60.9 %)	11 (68.8 %)	0.61
	frontal lobe	9 (39.1 %)	5 (31.3 %)	
multicentric disease	no	19 (82.6 %)	15 (93.8 %)	0.31
	yes	4 (17.4 %)	1 (6.3 %)	
grade 4 WHO 2016	G3	3 (13.0 %)	2 (12.5 %)	0.99
	G4	20 (87.0 %)	14 (87.5 %)	
IDH mutation	no	22 (95.7 %)	14 (87.5 %)	0.56
	yes	1 (4.3 %)	2 (12.5 %)	
extent of surgery	GTR	5 (21.7 %)	7 (43.8 %)	0.30
	STR	15 (65.2 %)	7 (43.8 %)	
	Biopsy	3 (13.0 %)	2 (12.5 %)	
total Gy	60	9 (39.1 %)	7 (43.8 %)	0.75
	50	12 (52.2 %)	9 (56.3 %)	
	40.05	2 (8.7 %)	0 (0.0 %)	
steroid dose 1st follow up	</=4mg	9 (69.2 %)	9 (81.8 %)	0.48
	>4mg	4 (30.8 %)	2 (18.2 %)	
progression in field	no	2 (8.7 %)	1 (6.3 %)	0.99
	yes	21 (91.3 %)	15 (93.8 %)	
progression out field	no	17 (73.9 %)	15 (93.8 %)	0.11
	yes	6 (26.1 %)	1 (6.3 %)	
T2/FLAIR edema volume	cm^3^	60.8 ± 60.61	38.6 ± 34.75	0.37
T2/FLAIR whole volume	cm^3^	80.6 ± 82.29	42.2 ± 37.48	0.15

TTP true tumour progression, PsP pseudoprogression, WHO world health organization, G grade, IDH isocitrate dehydrogenase, GTR gross total resection, STR subtotal resection, Gy gray, 1st first, mg milligrams, T2/FLAIR T2-weighted Fluid-Attenuated Inversion Recovery relapse edema and whole volume.

Categorical variables are reported as number (percentage). Continuous variables are reported as mean ± standard deviation.

**Table 1b:** Part 2. Differences between patients with psp and ttp.

		TTP (N=23)	PsP (N=16)	p
mgmt methylation	No	14 (60.9 %)	9 (56.3 %)	0.77
	Yes	9 (39.1 %)	7 (43.8 %)	
T1WCE volume cm^3^		19.8 ± 25.2111.7 (0.2–94.4)	3.6 ± 4.492.2 (0.1–13.9)	*<0.001*
platelets		212.7 ± 88.12209.0 (52.0–481.0)	199.7 ± 70.99196.5 (82.0–323.0)	0.62
neutrophils		5.9 ± 2.395.5 (1.9–10.1)	4.5 ± 2.554.1 (1.8–10.3)	0.061
lymphocytes		1.1 ± 0.560.8 (0.3–2.0)	1.4 ± 0.621.3 (0.5–2.5)	0.18
monocytes		0.7 ± 0.440.7 (0.0–2.0)	0.5 ± 0.220.5 (0.3–1.1)	0.51
NLR		7.3 ± 7.264.7 (2.6–36.1)	4.1 ± 3.722.8 (1.1–16.0)	*0.002*
SII		1511.0 ± 1615.33 890.5 (397.5–7538.9)	915.5 ± 1215.24 546.5 (152.0–5157.0)	*0.009*
SIRI		5.8 ± 8.173.3 (0.0–36.1)	2.1 ± 2.201.5 (0.7–9.9)	0.06

MGMT O6-Methylguanine-DNA methyltransferase), T1WCE T1-weighted contrast-enhanced MRI relapse volume, NLR Neutrophil-to-Lymphocyte Ratio, SII Systemic Immune-Inflammation Index, calculated as (Platelets x Neutrophils) / Lymphocytes, SIRI Systemic Inflammation Response Index, calculated as (Neutrophils x Monocytes) / Lymphocytes.

Categorical variables are reported as number (percentage). Continuous variables are reported as mean ± standard deviation.

### 3.1 Univariate analysis

In comparing PsP and TTP groups, our univariate analysis revealed that the neutrophil/lymphocyte ratio (NLR), systemic immune-inflammatory index (SII), and T1WCE volume were notably higher in patients with TTP.

Specifically, NLR values were 7.3 in TTP compared to 4.1 in PsP (p = 0.002), SII values were 1511.0 vs. 546.5 (p = 0.009), and T1WCE values were 19.8 cm^3 vs. 2.2 cm^3 (p < 0.001). Although absolute neutrophil counts and systemic inflammation response index (SIRI) were also higher in the TTP group, these differences did not achieve statistical significance.

These results are detailed in Table 1a, Table 1b.

### 3.2 Multivariate analysis

Our multivariate analysis differentiated two models: the “NLR Model” and the “SII Model.”.

In the NLR Model, both NLR (OR 7.9, 95% CI: 1.4 to 45.3, p = 0.020) and T1WCE volume (OR 3.0, 95% CI: 1.4 to 6.7, p = 0.007) were identified as significant predictors of TTP.

Similarly, the SII Model confirmed T1WCE volume (OR 2.7, 95% CI: 1.3 to 5.5, p = 0.006) and SII (OR 4.2, 95% CI: 1.1 to 15.3, p = 0.030) as significant predictors.

These findings underscore the predictive value of these markers in differentiating TTP from PsP, as elaborated in Table 2.

**Table 2:** Multivariate model of prognostic factors for TTP vs PsP.

	Multivariate Model NLROR (95 %CI), p value	Multivariate Model SII indexOR (95 %CI), p value
T1WCE volume (cm^3^)	3.02 (1.36–6.72); *0.007*	2.72 (1.34–5.51); *0.006*
NLR	7.92 (1.38–45.30); *0.020*	−
SII index	−	4.18 (1.14–15.27); *0.030*

### 3.3 Impact on overall survival

**Fig. 1:**
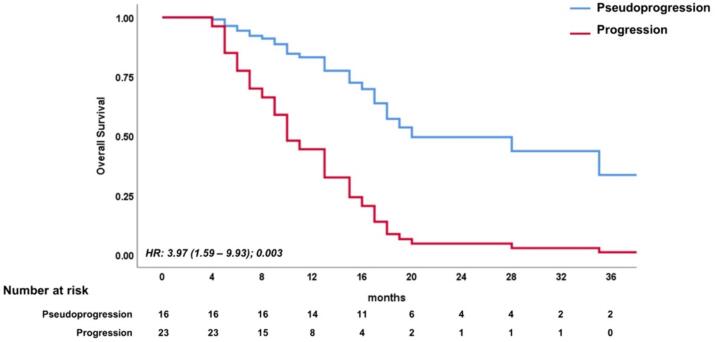
The Kaplan-Meier curve.

Our survival analysis indicated a significantly poorer overall survival (OS) in patients with TTP compared to those with PsP (Fig. 1).

The hazard ratio (HR) for TTP was 3.97 (95% CI: 1.59 to 9.93, p = 0.003).

However, when considering other factors in the multivariate model, only the distinction between progression and pseudoprogression remained a significant predictor of OS, as shown in Table 3.

**Table 3:** Effect of different factors on the time to death according to univariable and multivariable Cox regression models.

	Univariable		Multivariable	
	HR (95 % CI)	p-value	HR (95 % CI)	p-value
TTP vs PsP	4.41 (1.90–10.24)	*0.001*	3.97 (1.59–9.93)	*0.003*
T1WCE volume (cm^3^)	1.27 (1.02–1.59)	*0.033*	1.07 (0.85–1.34)	0.56
NLR	1.41 (0.88–2.26)	0.15		
SII index	1.27 (0.87–1.87)	0.22		

TTP true tumour progression, PsP pseudoprogression, OR odds ratio, NLR neutrophils/lymphocytes ratio, SII Systemic immune-inflammation index, T1WCE volume of enhancing tissue on post-contrast T1w MRI images.

TTP true tumour progression, PsP pseudoprogression, HR hazard ratio, NLR neutrophils/lymphocytes ratio, SII Systemic immune-inflammation index, T1WCE Volume of enhancing tissue on post-contrast T1w MRI images.

### 3.4 Diagnostic accuracy and optimal thresholds

The ROC curve and AUC analysis provided the thresholds for the three variables.

For NLR, the threshold was 3.18 with a specificity of 75.0% and a sensitivity of 87.0%. For SII, it was 620.55 with a specificity of 68.8% and a sensitivity of 82.6%. Lastly, for hyperintensity volume on T1- weighted contrast-enhanced the threshold was 5.00 cm3 with a specificity of 81.3% and a sensitivity of 78.3%.

**Fig. 2:**
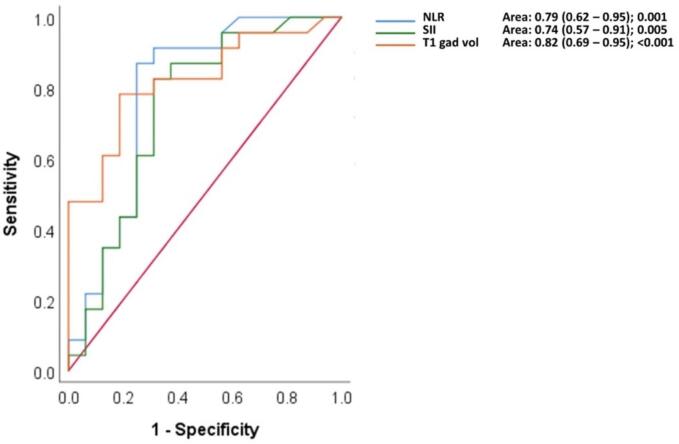
The ROC curve and AUC. Cut-off. NLR: 3.18 (Specificity: 75.0 %; Sensitivity: 87.0 %). SII: 620.55 (Specificity: 68.8 %; Sensitivity: 82.6 %). Volume of enhancing tissue on post-contrast T1w: 5.00 (Specificity: 81.3 %; Sensitivity:78.3 %).

These thresholds, depicted in Fig. 2, are instrumental in enhancing the diagnostic accuracy of our predictive model. The comparison between the 3 curves was not significant.

## 4 Discussion

This study aimed to delineate clear markers for differentiating true tumor TTP from PsP in patients with HGGs [7], [8] within the critical early period of the first three months post-radiotherapy, extending to a maximum of six months [5].

This distinction is pivotal for effective patient management, particularly during this early and often ambiguous phase where treatment responses are most variable [37], [38], [39].

All patients were re-evaluated by a team of experienced neuroradiologists, ensuring a consistent and uniform distinction between PsP and TTP, thereby minimizing the risk of misclassification, particularly false negatives among PsP cases [12], [40].

Our findings highlight the significant role of systemic inflammation markers and T1WCE volume in this differentiation within this timeframe, which aligns with and expands upon the existing body of research [7].

A significant strength of our study is the comprehensive consideration of various clinical data often associated with worse prognosis and early true tumor progression (TTP) post-treatment. Specifically, we took into account critical factors such as the absence of the IDH mutation, the absence of MGMT methylation, and the increased use of corticosteroids in the early post-treatment follow-up. These factors are well-documented in the literature for their impact on patient outcomes and provide a robust framework for distinguishing TTP from PsP. By incorporating these variables into our analysis, we were able to enhance the accuracy and relevance of our findings, contributing valuable insights to the existing body of knowledge on high-grade gliomas.

The main limitation of our case series was the small cohort size. However, this reduction in patient numbers was due to our stringent inclusion criteria, which aimed to ensure data accuracy and relevance. Patients were excluded for reasons such as incomplete follow-up data, lack of consistent imaging, and non-compliance with the WHO 2021 classification criteria. While this exclusion could suggest a selection bias, it was necessary to maintain the integrity and reliability of the study. Nonetheless, the consistency and pertinence of our findings offer a substantial contribution to the existing literature.

While the exclusion of advanced imaging sequences might be viewed as another constraint, it’s noteworthy that such sequences have not demonstrated significant diagnostic strength, particularly within the crucial initial three months of follow-up [12].

Our focus, therefore, was deliberately tailored to assess the role of inflammatory indices and volumetric analysis as per the framework outlined in La Fevre’s review [7], [8].

In the literature, in line with our study, the few papers that considered the neutrophil/lymphocyte ratio (NLR) to distinguish between PD and PsP had promising results, suggesting its potential as a reliable marker [22]. While studies analyzing the role of the systemic inflammation response index (SIRI) and systemic immune-inflammation index (SII) post-radiotherapy are lacking, significant results have been observed at other timings. For instance, a low SIRI preoperatively is associated with better survival [15], and a high SII at diagnosis correlates with worse survival [17], [21]. These findings suggest that inflammatory indices hold prognostic value and could potentially aid in early post-treatment assessments.

Our results also focus on the volume of enhancing tissue on post-contrast T1 weighted (T1WCE), measured in cm^3^ on the MRI post-RT, in line with what was hypothesized in a part of the phase III SpectroGlio trial (NCT01507506) [5] and is particularly relevant in light of the new RANO 2.0 classification [41]. According to these updated guidelines, the incidence of pseudoprogression is significantly high in the first 12 weeks post-chemoradiotherapy for glioblastomas [12], [38], [41] and may extend beyond 3 months for IDH-mutated gliomas and other glial tumours [42]. Especially during the first 12 weeks for glioblastomas, the correlation between radiological changes and true progression, as well as survival, is poorly defined. Consequently, RANO 2.0 proposes that for clinically stable patients showing signs of radiological progression, MRI should be repeated at 4- or 8-week intervals to confirm progression prior to any significant changes in the patient’s treatment plan [38], [41].

This recommendation resonates with our observation that the relationship between pre-existing volumes and volumes assessed after radiotherapy may not be as crucial as previously thought.

Our results indeed indirectly confirm that the first post-RT MRI can serve as an independent and reliable basis for response assessment, regardless of the initial disease presentation [38].

The timing and aim of our study are therefore highly relevant and underline the need for continued research and validation of diagnostic markers that can help to define radiological response in the first 3–6 months after the end of radiotherapy.

## 5 Conclusion

Our study underscores the potential of systemic inflammation markers and volume of enhancing tissue on post-contrast T1 weighted (T1WCE) MRI sequences, cost-effective tools for differentiating between TTP and PsP in the early post-radiotherapy period.

By integrating these markers into the clinical decision-making process, we can enhance the accuracy of early treatment assessments, thereby improving patient management and outcomes in HGGs cases. However, it is important to note that our results should be confirmed with a broader casuistry to solidify these findings and ensure their applicability in diverse clinical settings.

## Funding information

Network Programme All-Ages Malignant Glioma: Holistic Management In The Personalised Minimally-Invasive Medicine Era – From Lab To Rehab – GLI-HOPE.

## Declaration of competing interest

The authors declare that they have no known competing financial interests or personal relationships that could have appeared to influence the work reported in this paper.
